# Androgen receptor expression in normal breast tissue and subsequent breast cancer risk

**DOI:** 10.1038/s41523-018-0085-3

**Published:** 2018-09-21

**Authors:** Kevin H. Kensler, Francisco Beca, Gabrielle M. Baker, Yujing J. Heng, Andrew H. Beck, Stuart J. Schnitt, Aditi Hazra, Bernard A. Rosner, A. Heather Eliassen, Susan E. Hankinson, Myles Brown, Rulla M. Tamimi

**Affiliations:** 1 Department of Medical Oncology, Dana-Farber Cancer Institute, Boston, MA 02215 USA; 2 Department of Epidemiology, Harvard T.H. Chan School of Public Health, Boston, MA 02115 USA; 3 Department of Pathology, Stanford University School of Medicine, Stanford, CA 94035 USA; 4 Department of Pathology, Beth Israel Deaconess Medical Center, Boston, MA 02215 USA; 5 Harvard Medical School, Boston, MA 02215 USA; 6 PathAI, Cambridge, MA 02141 USA; 7 Department of Pathology, Brigham and Women’s Hospital, Boston, MA 02115 USA; 8 Division of Preventive Medicine, Department of Medicine, Brigham and Women’s Hospital, Boston, MA 02115 USA; 9 Channing Division of Network Medicine, Department of Medicine, Brigham and Women’s Hospital and Harvard Medical School, Boston, MA 02115 USA; 10 Department of Biostatistics, Harvard T.H. Chan School of Public Health, Boston, MA 02115 USA; 11 Department of Biostatistics and Epidemiology, University of Massachusetts School of Public Health and Health Sciences, Amherst, MA 01003 USA

## Abstract

Sex steroid hormone signaling is critical in the development of breast cancers, although the role of the androgen receptor remains unclear. This study evaluated androgen receptor (AR) expression in normal breast tissue as a potential marker of breast cancer risk. We conducted a nested case–control study of women with benign breast disease (BBD) within the Nurses’ Health Studies. Epithelial AR expression was assessed by immunohistochemistry in normal tissue from the BBD biopsy and the percent of positive nuclei was estimated in ordinal categories of 10% for 78 breast cancer cases and 276 controls. Logistic regression models adjusting for the matching factors and BBD lesion type were used to calculate odds ratios (ORs) for the association between AR expression (tertiles: ≤10%, 11–30%, and >30%) and breast cancer risk. AR expression in normal breast tissue was not associated with subsequent breast cancer risk (OR_T3vsT1_ = 0.9, 95% CI = 0.4–1.8, *p* trend = 0.68). In comparison with low AR/low ER women, ORs of 0.4 (95% CI = 0.1–1.2) for high AR/high ER women, 1.8 (95% CI = 0.4–7.8) for low AR/high ER women, and 0.7 (95% CI = 0.3–1.6) for high AR/low ER women were observed (*p* interaction = 0.21). Ki67 did not modify the association between AR expression and breast cancer risk (*p* interaction = 0.75). There was little evidence for an overall association between AR expression in normal breast tissue and breast cancer risk. These findings did not show that the AR association varied by Ki67 expression in normal breast tissue, though there was suggestive heterogeneity by ER expression.

## Molecular epidemiology: Male hormone receptor expression not linked to cancer risk

Levels of male hormone receptors in normal breast tissue do not seem to impact a woman’s risk of developing breast cancer. Kevin Kensler from the Dana-Farber Cancer Institute in Boston, Massachusetts, USA, and colleagues quantified expression of the androgen receptor (AR) in biopsy tissue taken from 354 women with benign breast disease, 78 of whom were later diagnosed with breast cancer. Although AR levels have been linked to outcomes in women who already have breast tumors, the study authors found no such association between AR expression and disease risk while women were still cancer-free. There was also little evidence for an interaction between AR levels and those of the estrogen receptor or the proliferation marker Ki67 on the incidence of breast cancer. However, the researchers caution, larger studies are needed to verify the results.

## Introduction

Sex steroid hormones play a critical role in the development and progression of breast cancers.^1,2^ While estrogen signaling has long been hypothesized to be a promoter of breast carcinogenesis,^3,4^ the effects of androgen signaling are unclear. Circulating androgens are associated with breast cancer risk among postmenopausal women, conferring roughly twofold higher risk comparing highest-to-lowest quintiles.^5^ Some of this apparent association may be due to the conversion of androgens to estrogens, leading to activation of estrogen signaling pathways in the breast. When circulating androgens are adjusted for circulating estradiol, these associations attenuate, but not entirely to unity, suggesting a potential effect of androgens on the breast, independent of their conversion to estrogens.^5–7^ Circulating hormones show moderate correlations with hormone levels in the breast, indicating that these may be indirect markers of the hormonal milieu in the breast.^8–12^

The androgen receptor (AR) is expressed in 60–80% of breast cancers and is an emerging prognostic and predictive marker, as well as potential therapeutic target, in breast cancer.^13,14^ AR is more commonly co-expressed in estrogen receptor-positive (ER+) breast cancers (70–90%), though is still expressed in 40% of ER− cancers.^15–17^ Evidence from in vitro models suggests that the effects of AR signaling in breast cancers may depend on tumor ER expression.^18,19^ AR signaling promotes tumor growth and inhibits apoptosis in ER− breast cancers,^20^ while antagonizing ER signaling pathways in ER+ breast cancers.^21^ Epidemiologic studies have consistently found that AR expression is associated with improved breast cancer prognosis for women with ER+ breast cancer, in concordance with in vitro evidence.^22^ However, results from epidemiologic studies among women with ER− breast cancer are quite mixed.^22^ Although AR is expressed in normal breast epithelium, to date, AR expression and its potential interaction with ER expression have not been evaluated in normal breast tissue with respect to breast cancer risk. Few markers in normal tissue have been identified that predict future breast cancer risk.^23–26^ Of these, Ki67, a nuclear protein present in active phases of the cell cycle,^27^ has been most consistently linked to breast cancer risk.^28–30^ This association may be limited to women with low ER expression.^29^

In this analysis, we evaluated AR expression in normal breast tissue as a potential predictor of subsequent breast cancer risk in a nested case–control study within the Nurses’ Health Study and Nurses’ Health Study II cohorts and whether this association differed by ER co-expression. We further assessed potential effect modification by Ki67, given the heterogeneity previously observed for the Ki67-breast cancer association by ER expression. Finally, we explored the relationships between established breast cancer risk factors and AR expression in normal tissue.

## Results

The median AR expression was 20% (IQR: 10%–43%) in this population of 354 women (78 cases and 276 controls). The Spearman correlations were 0.44 between AR and ER expression and −0.09 between AR and Ki67 expression. Age-standardized distributions of the study matching factors and BBD lesion type are shown in Table 1. The time elapsed between BBD biopsy and the index date was suggestively longer for controls. Cases were more likely to have proliferative BBD lesions with atypia than controls.

**Table 1:** Age-standardized distributions of study matching factors and BBD lesion type among 78 breast cancer cases and 276 controls

Variable	Breast cancer cases (*n* = 78)	Controls (*n* = 276)
Age at cancer diagnosis, mean (SD)	53.9 (8.7)	—
Year of BBD biopsy, %		
Before 1980	41	40
1980–1989	48	46
After 1989	11	15
Time from BBD biopsy to index date, %		
0.5–4.9 years	26	48
5.0–9.9 years	39	24
10.0–14.9 years	20	20
15.0+ years	15	8
BBD lesion type, %		
Non-proliferative	26	29
Proliferative without atypia	48	57
Atypical hyperplasia	26	15

In logistic regression models adjusting for the matching factors, AR expression in normal breast tissue was not associated with subsequent breast cancer risk (Table 2). After further adjustment for BBD lesion type, women with >30% AR expression had a non-significant 10% decrease in breast cancer risk compared to women with <10% expression (OR = 0.9, 95% CI = 0.4–1.8, *p* trend = 0.68). Similarly, a 10% increase in AR expression was not associated with breast cancer risk (OR = 1.0, 95% CI = 0.9–1.1) in the fully adjusted model.

**Table 2:** Odds ratios (95% confidence interval) of developing breast cancer by tertile and per each 10% increase in AR expression in normal breast tissue

	Tertile 1 (≤10%)	Tertile 2 (11–30%)	Tertile 3 (>30%)	*P* trend	10% Increase
Cases/controls	19/63	31/108	28/105		78/276
Model 1	Ref	1.0 (0.5–1.9)	1.0 (0.5–2.0)	0.96	1.0 (0.9–1.1)
Model 2	Ref	1.0 (0.5–1.9)	0.9 (0.4–1.8)	0.68	1.0 (0.9–1.1)

Model 1: Adjusting for age at breast cancer diagnosis/index date (<45, 45–54, and 55+), year of the BBD diagnosis (before 1980, 1980–1989, 1990 or later), and years elapsed between BBD diagnosis and breast cancer diagnosis/index date (0.5–4.9, 5–9.9, 10–14.9, and 15.0+)

Model 2: Adjusted for covariates in Model 1 + BBD lesion type (non-proliferative, proliferative without atypia, and atypical hyperplasia)

**Fig. 1:**
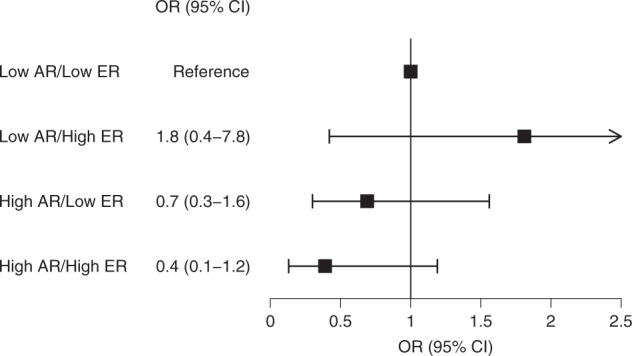
Odds ratios (95% confidence interval) of developing breast cancer by cross-classified AR and ER expression in normal breast TDLUs. ORs are estimated from unconditional logistic regression models among 47 cases and 127 controls and are adjusted for age at cancer diagnosis/index date, year of BBD biopsy, time between BBD diagnosis and breast cancer diagnosis/index date, and BBD lesion type. AR and ER are dichotomized at their median levels (20% for AR and 10% for ER). *P* value for test of heterogeneity is 0.21

Among the 47 cases and 127 controls with measured AR and ER expression, there was no significant heterogeneity of the association between AR expression and the incidence of breast cancer by co-expression of ER in normal tissue (*p* heterogeneity = 0.21) (Fig. 1). Compared to women with low AR/low ER expression, women with high AR/high ER expression had a non-significant 60% lower risk of breast cancer (OR = 0.4, 95% CI = 0.1–1.2). Likewise, there was suggestive non-significant lower risk for women with high AR/low ER expression (OR = 0.7, 95% CI = 0.3–1.6), and higher risk for women with low AR/high ER expression (OR = 1.8, 95% CI = 0.4–7.8).

**Fig. 2:**
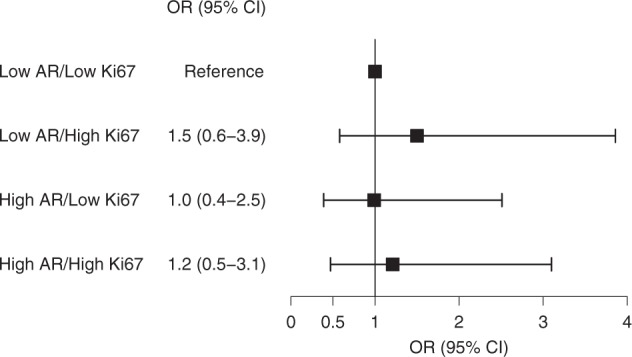
Odds ratios (95% confidence interval) of developing breast cancer by cross-classified AR and Ki67 expression in normal breast TDLUs. ORs are estimated from unconditional logistic regression models among 57 cases and 195 controls and are adjusted for age at cancer diagnosis/index date, year of BBD biopsy, time between BBD diagnosis and breast cancer diagnosis/index date, and BBD lesion type. AR and Ki67 are dichotomized at their median levels (20% for AR and 4% for Ki67). *P* value for test of heterogeneity is 0.75

Likewise, there was no significant effect modification by Ki67 expression (*p* heterogeneity = 0.75) (Fig. 2) among the 57 cases and 195 controls with measured AR and Ki67 expression. Women with Ki67 expression above the median had suggestively higher risk of breast cancer, irrespective of AR expression (low AR/high Ki67: OR = 1.5, 95% CI = 0.6–3.9; high AR/high Ki67: OR = 1.2, 95% CI = 0.5–3.1), relative to women with low AR and low Ki67 expression. High AR and low Ki67 expression was not associated with breast cancer risk, in comparison to low AR and low Ki67 expression (OR = 1.0, 95% CI = 0.4–2.5).

Exploratory analyses regarding the relationship between breast cancer risk factors and AR expression in normal tissue yielded few apparent associations (Table 3). AR expression non-significantly increased with age. Mean AR expression in normal tissue was higher in women with atypical hyperplastic BBD lesions (mean 22.1 [95% CI = 14.3–34.3]) compared to women with proliferative lesions without atypia (mean 15.2 [95% CI = 12.2–18.8]) or women with non-proliferative lesions (mean 13.4 [95% CI = 9.9–18.1]), though these differences did not achieve statistical significance (*p* value = 0.18 from age-adjusted ANCOVA). No other notable associations were observed with breast cancer risk factors.

**Table 3:** Breast cancer risk factors at time of BBD biopsy in relation to age-adjusted mean AR expression (95% confidence interval) in normal TDLUs among 276 controls

	*n*	Mean AR expression (95% CI)	*P* -value^a^
Age at BBD biopsy (yr.)^b^			0.15
<40	62	12.7 (9.0–17.8)	
40–50	147	15.6 (12.5–19.4)	
>50	67	18.1 (13.0–25.1)	
BBD lesion type			0.18
Non-proliferative	82	13.4 (9.9–18.1)	
Proliferative without atypia	155	15.2 (12.2–18.8)	
Atypical hyperplasia	39	22.1 (14.3–34.3)	
Age at first birth			0.51
Nulliparous	12	15.8 (7.3–34.0)	
<25 years	147	16.0 (12.9–20.0)	
25–29 years	91	14.8 (11.2–19.6)	
30+ years	13	8.8 (4.2–18.5)	
Age at menarche (yr.)			0.33
<12	56	13.9 (9.7–19.9)	
12	79	15.9 (11.8–21.5)	
13	80	13.9 (10.3–18.7)	
14+	59	19.3 (13.6–27.3)	
Age at menopause (yr.)			0.82
Premenopausal	122	17.7 (11.3–19.1)	
<50	81	16.8 (12.3–23.0)	
50+	59	16.2 (11.3–23.2)	
Menopausal hormone therapy use^c^			0.74
Ever	71	17.5 (12.6–24.3)	
Never	70	16.2 (11.6–22.5)	
Oral contraceptive use			0.18
Ever	131	14.0 (11.1–17.8)	
Never	140	17.6 (14.0–22.1)	
BMI (kg/m^2^) at BBD biopsy			0.53
<25.0	179	16.0 (13.1–19.5)	
25.0–29.9	57	15.3 (10.7–21.9)	
30.0+	37	13.7 (8.8–21.3)	
BMI (kg/m^2^) at age 18			0.51
<19.0	58	11.7 (8.3–16.5)	
19.0–24.9	160	18.3 (14.9–22.5)	
25.0+	26	13.9 (8.3–23.3)	
Weight change since age 18			0.77
Gain <2 kg	46	14.9 (10.1–21.9)	
Gain 2–20 kg	100	17.8 (13.7–23.2)	
Gain 20+ kg	108	15.1 (11.8–19.5)	
Alcohol consumption (g/week)			0.39
None	81	17.4 (13.0–23.4)	
0.1–4.9	75	13.0 (9.5–17.6)	
5.0–14.9	38	19.8 (12.9–30.3)	
15.0+	21	19.3 (10.9–34.3)	
Family history of breast cancer			0.19
Yes	44	19.7 (13.2–29.4)	
No	232	14.7 (12.4–17.5)	

Analyses are restricted to individuals with non-missing data. AR expression was log-transformed for analysis of covariance

^a^*P* -values are from global test of heterogeneity or test for trend (age at BBD biopsy, age at menarche, BMI at BBD biopsy, BMI at age 18, weight change, and alcohol consumption)

^b^Not adjusted for age

^c^Restricted to postmenopausal women

## Discussion

The androgen receptor is an emerging biomarker of interest in breast cancer. Prior studies have shown that AR may have utility as a predictive or prognostic marker, or may even be a viable therapeutic target for certain breast cancer subtypes.^13,14^ In a nested case–control study within the NHS and NHSII cohorts, we found no association between AR expression in normal TDLUs and the subsequent incidence of breast cancer.

We likewise found little evidence for an interaction between AR and ER expression in normal breast TDLUs with respect to the incidence of breast cancer. However, in vitro and population evidence support the presence of a biologic interaction between AR and ER signaling in breast cancers.^18,19^ In ER− breast cancers, AR signaling can stimulate tumor growth. In contrast, in ER+ breast cancers, AR may have anti-proliferative effects—antagonizing ER signaling through the occupancy of shared or distinct-binding sites at ER target genes or through competition for transcriptional co-regulators, abrogating their interaction with ER.^18,19^ Comparatively little is known about the interactions between AR and ER signaling in normal breast epithelium, though evidence from animal models suggests that the AR may still inhibit estrogen-stimulated proliferation in normal breast tissue.^31–33^ We observed that women with high AR and high ER expression had suggestively lower risk of breast cancer, though this did not achieve statistical significance. While there were few individuals with low AR and high ER, they experience apparent higher risk of breast cancer, though again this was not statistically significant. The AR is more commonly expressed in breast epithelial cells than the ER, and while co-localization of AR and ER can occur, more often AR is expressed without ER.^21^ ER infrequently co-localizes with Ki67 in normal breast tissue, though this does often occur in malignant tissue.^34,35^ Prior analyses in this case–control study indicated that women with high Ki67 and low ER expression in normal TDLUs experienced higher breast cancer risk.^29^ Our findings indicated no heterogeneity for the association between Ki67 expression and breast cancer risk with respect to AR expression.

Circulating androgens are a consistent risk factor for breast cancer among postmenopausal women. Additionally, androgen levels in plasma were shown to be correlated with several breast cancer risk factors including age, menopausal status, obesity, smoking, and alcohol consumption in a pooled analysis of 13 prospective studies.^36^ This analysis showed no link between other reproductive factors (such as age at menarche, age at first birth, and parity) and concentrations of circulating androgens. A prior analysis in the NHS found that positive associations between obesity and weight change and breast cancer risk, and the inverse association between physical activity and breast cancer risk, were generally stronger for the risk of AR− tumors.^37^ Age at first birth and oral contraceptive use were also found to be associated with the incidence of AR− breast cancer.^38^ In contrast, alcohol consumption was found to be associated with the incidence of AR+ tumors.^39^ Apart from a non-significant suggestive positive association with age, we found no relationships between these risk factors and AR expression levels in normal breast tissue. Prior studies have evaluated the associations between breast cancer risk factors and ER and progesterone receptor (PR) expression in normal tissue.^26,40–42^ These studies found age, adult BMI, and alcohol consumption to be positively associated with ER expression and an inverse association with breastfeeding among parous women. Age, height, adult BMI, and BMI at age 18 were correlated with higher PR expression. While these risk factors may affect hormone receptor signaling in normal tissue, we found no relationship between these variables and AR expression, suggesting that their effects may not be through AR signaling pathways.

This study benefits from a unique design that allowed for the evaluation of a hypothesis that AR signaling in normal tissue may contribute to carcinogenesis in the breast. Given the uniqueness and resources required of the design, however, the sample size is quite limited. This reduced the statistical power available, in particular to evaluate interactions between AR and ER and Ki67 expression. Notably, this sample size has been sufficient to observe significant associations between IGF1R, EZH2, and Ki67 and breast cancer risk within the same nested case–control study.^23–25,29^ The limited sample size also precludes adjustment for many variables, leaving the findings susceptible to possible residual confounding. Additionally, the success rate for acquiring tissue from the BBD biopsy was low; though the women for whom we successfully obtain or do not obtain tissue have similar risk factor profiles (Supplementary Table 1). Thus, there is lesser potential for a selection bias through this mechanism. Measurement error in the assessment of tissue markers could arise through the use of TMAs rather than whole tissue sections. However, the ICCs across cores indicated good reliability for AR and ER expression, and modest for Ki67, suggesting that the cores may sufficiently represent all normal breast TDLUs. Finally, there was an apparent association between type of BBD lesion and AR expression, suggesting potential field effects from the BBD lesion in the normal TDLUs included in the TMAs. Hence, our findings may only be applicable to women with BBD.

In conclusion, we found no association between AR expression in normal breast TDLUs and subsequent breast cancer risk, nor any evidence of effect heterogeneity by co-expression of ER or Ki67. Due to the limited sample size in our analysis, these associations should be further evaluated in other populations.

## Methods

### Study population

The Nurses’ Health Study (NHS) was established in 1976 with the enrollment of 121,700 US female registered nurses aged 30–55 years, while the Nurses’ Health Study II (NHSII) consists of 116,429 US female registered nurses aged 25–42 years at cohort baseline in 1989. Detailed descriptions of cohort procedures have been reported elsewhere.^43^ Briefly, cohort members completed baseline questionnaires that provided medical histories and extensive information about demographic, lifestyle, reproductive, and dietary risk factors for breast cancer. Cohort members have updated this information biennially through follow-up questionnaires. NHS and NHSII participants also report new diagnoses of breast cancer and fibrocystic or benign breast disease (BBD) in the biennial questionnaires. These diagnoses are confirmed via medical record review or verbal confirmation from the nurse herself. All study protocols were approved by the institutional review board at Brigham and Women’s Hospital (Boston, MA) and informed consent was obtained from all study participants.

A nested case–control study was constructed within the two cohorts, wherein cases were women who reported a diagnosis of breast cancer after cohort baseline (through 1998 for NHS and 1999 for NHSII) and had previously reported a BBD diagnosis (either prior to study entry or after study baseline). Controls were women with a diagnosis of BBD who did not subsequently develop breast cancer. Details of this case–control study have been described elsewhere.^23,44^ A median of 8 years (interquartile range [IQR] 5–12) elapsed between the BBD and breast cancer diagnoses for cases, and cases for which this time period was <6 months were excluded. Cases and controls were matched 1:4 on the age at breast cancer diagnosis (index date for controls), year of the BBD diagnosis, and years elapsed between BBD diagnosis and breast cancer diagnosis (index date).

### BBD tissue acquisition and immunohistochemical assays

For the identified cases and controls, study investigators requested H&E slides and later formalin-fixed paraffin-embedded tissue from the BBD biopsy from the diagnosing hospital. Slides were obtained for 463 cases and 1853 controls, and then within this group BBD tissue blocks were obtained for 177 cases and 719 controls. Three hundred eighty-eight participants with the following BBD lesion types were eligible for inclusion in tissue microarrays (TMAs): apocrine metaplasia, non-apocrine cysts, usual ductal hyperplasia, atypical ductal hyperplasia, and atypical lobular hyperplasia. For each of these participants, breast pathologists selected three representative areas of tissue containing normal terminal ductal lobular units (TDLUs).^45^ These areas were cored (0.6 mm) and assembled into six TMAs. The TMAs were then sectioned and stained for AR protein expression (Dako M3562 antibody, 1:200) and four of six TMAs for ER protein expression (Neomarkers RM-9101-S antibody, 1:40). The stained TMAs were then reviewed by pathologists who estimated AR and ER expression as 0%, 1%, or in ordinal categories of 10% in the epithelial tissue in each core. The mean expression for AR and ER was then calculated across the three cores for each individual. The maximum expression and geometric mean across cores were also evaluated and yielded similar results (data not shown). Ki67 expression (Vector VP-RM04 antibody, 1:250) was scored using Definiens Tissue Studio (Munich, Germany), an automated imaging analysis software program, which produced a 0–100% continuous estimate of expression. Women with low cellularity (<100 detected nuclei) for Ki67 expression were excluded from the analysis. Intraclass correlations were 0.66 for AR, 0.69 for ER, and 0.36 for Ki67, indicating some heterogeneity in expression across cores within individuals. Thirty-four individuals did not have evaluable AR expression in any of the three cores, leaving to a final sample size of 354 individuals (78 cases and 276 controls). Characteristics of participants selected into the nested case–control study with and without evaluable AR expression in normal tissue are shown in the supplement.

### Statistical analysis

AR expression was categorized into tertiles as defined by the distribution among the controls (0–10%, 11–30%, and >30%). As tissue from the BBD biopsy was not obtained from all members of case–control sets, unconditional logistic regression models adjusting for the study matching factors and type of BBD lesion were used to estimate odds ratios (OR) and 95% confidence intervals (95% CI) for the association between AR expression in normal breast tissue and subsequent breast cancer risk. Other established breast cancer risk factors were considered as potential confounders, but were not included in final models, given their weak associations with AR expression and the limited sample size. Tests for linear trends were performed by fitting the regression models with a linear covariate taking the median AR expression level of each tertile. To evaluate the potential interaction between AR and ER or Ki67 expression, four categories were created by dichotomizing each distribution at the median level among the controls (20% for AR, 10% for ER, and 4% for Ki67). The *p* value for the test of heterogeneity was then calculated using a likelihood ratio test for the AR-by-ER or AR-by-Ki67 product term. The associations between established breast cancer risk factors and AR expression (natural log-transformed) among controls were assessed using analysis of covariance (ANCOVA) adjusting for age at BBD biopsy. Nineteen controls (of 276) with 0% AR expression were reassigned to 1% (minimum detectable positive AR expression) for the ANCOVA analysis. A 0.05 level of significance was used for all statistical tests. All analyses were performed using SAS version 9.4 (SAS Institute, Cary, NC). As applicable, the analyses performed and reported are adherent to the REMARK recommendations for tumor marker prognostic studies.^46^

## Electronic supplementary material

Supplemental Table 1

## Data Availability

The data that support the findings of this study are available from the Nurses’ Health Studies, however they are not publicly available. Investigators interested in using the data can request access, and feasibility will be discussed at an investigators meeting. Limits are not placed on scientific questions or methods, and there is no requirement for co-authorship. Additional data sharing information and policy details can be accessed at http://www.nurseshealthstudy.org/researchers.

## Electronic supplementary material

**Supplementary information** accompanies the paper on the *npj Breast Cancer* website (10.1038/s41523-018-0085-3).
