# Supplementary material for: Androgen receptor expression in normal breast tissue and subsequent breast cancer risk
Source: NPJ Breast Cancer. 2018 Sep 21;4:33. doi: 10.1038/s41523-018-0085-3 (PMC6155011; doi:10.1038/s41523-018-0085-3)
Supplement: Supplementary file 1 — Supplemental Table 1 [file 41523_2018_85_MOESM1_ESM.docx]

**Supplementary Table 1**. Age-standardized distributions of breast cancer risk factors comparing individuals selected into case-control study with and without evaluable AR expression^1^

|  | Evaluable AR  (n=354) | No Evaluable AR  (n=2,118) |
| --- | --- | --- |
| Age at cancer diagnosis/index date^2^ | 52.2 (8.6) | 54.2 (8.8) |
| Year of BBD biopsy, % |  |  |
| Before 1980 | 41 | 44 |
| 1980-1989 | 46 | 37 |
| After 1989 | 13 | 18 |
| Age at BBD biopsy, % |  |  |
| <40 years | 20 | 31 |
| 40-50 years | 51 | 42 |
| >50 years | 28 | 26 |
| BBD category, % |  |  |
| Non-proliferative | 26 | 34 |
| Proliferative without atypia | 56 | 54 |
| Atypical hyperplasia | 18 | 12 |
| Age at first birth, % |  |  |
| Nulliparous | 4 | 6 |
| <25 years | 50 | 47 |
| 25-29 years | 37 | 33 |
| 30+ years | 5 | 10 |
| Missing | 4 | 4 |
| Age at menarche (yr.) , % |  |  |
| <12 | 22 | 21 |
| 12 | 30 | 26 |
| 13 | 28 | 31 |
| 14+ | 20 | 22 |
| Missing | 1 | 0 |
| Age at menopause (yr.) , % |  |  |
| Premenopausal | 38 | 33 |
| <50 | 32 | 36 |
| 50+ | 25 | 26 |
| Missing | 5 | 5 |
|  |  |  |
|  |  |  |
| Postmenopausal hormone therapy use, % |  |  |
| Ever | 32 | 40 |
| Never | 28 | 23 |
| Premenopausal | 40 | 36 |
| Missing | 0 | 1 |
| Oral contraceptive use, % |  |  |
| Ever | 46 | 52 |
| Never | 52 | 47 |
| Missing | 2 | 2 |
| BMI (kg/m^2^) at BBD biopsy, % |  |  |
| <25.0 | 64 | 61 |
| 25.0-29.9 | 23 | 26 |
| 30.0+ | 13 | 13 |
| BMI at age 18 years, % |  |  |
| <19.0 | 20 | 20 |
| 19.0-24.9 | 59 | 62 |
| 25.0+ | 8 | 5 |
| Missing | 14 | 13 |
| Weight change since age 18, % |  |  |
| Gain <2 kg | 18 | 16 |
| Gain 2-10 kg | 34 | 33 |
| Gain 10+ kg | 40 | 43 |
| Missing | 8 | 8 |
| Alcohol consumption (g/week), % |  |  |
| None | 37 | 35 |
| 0.1-4.9 | 34 | 38 |
| 5.0-14.9 | 20 | 20 |
| 15.0+ | 8 | 8 |
| Family history of breast cancer, % |  |  |
| Yes | 18 | 19 |
| No | 82 | 81 |

^1^ Values are means(SD) or percentages and are standardizaed to the age distribution of the study population. Values of polytomous variables may not sum to 100% due to rounding

^2^ Value is not age-adjusted.
